# Lifecourse Factors Influencing the Quality of Life of African American Parent-Adult Daughter Dementia Dyads

**DOI:** 10.1093/geroni/igaf122.2074

**Published:** 2025-12-31

**Authors:** Kalisha Bonds Johnson, Kenneth Hepburn, Wizdom Powell, Karen Lyons

**Affiliations:** Emory University, Atlanta, Georgia, United States; Emory University, Atlanta, Georgia, United States; University of Connecticut School of Medicine, Farmington, Connecticut, United States; Boston College, Chestnut Hill, Massachusetts, United States

## Abstract

African American adult daughters are the most common care partners of African American persons living with dementia. Yet little is known about how these African American parent-adult daughter dementia dyads (i.e., parent living with dementia and their adult daughter care partner) navigate this disease process or how their quality of life (QOL) is influenced. Guided by the NIA Health Disparities Research Framework, Black Family Social-Ecological Context Model, and Superwoman Schema, this presentation identifies lifecourse factors (e.g., behavioral, sociocultural, and environmental) significantly associated with the quality of life of these dyads. In this sample of 39 African American parent-adult daughter dementia dyads, data were analyzed using multilevel modeling to control for the interdependence of the data. On average, the parents’ QOL did not significantly differ from the adult daughters’ QOL. Parents experienced significantly better QOL when the adult daughters reported a more positive family decision-making process but experienced significantly worse QOL when the adult daughters reported greater cultural justification for caregiving. Adult daughters experienced significantly better QOL when they reported greater family social support and had a more positive family decision-making process. However, adult daughters reported significantly worse QOL when their parents had appointment with healthcare professionals within 30 days of the study visit compared to those parents who did not. Findings suggest taking a lifecourse perspective may hold promise in improving the QOL of African American parent-adult daughter dementia dyads. Findings will be discussed considering this first phase of an explanatory sequential mixed methods intervention design.

